# Medical and nursing clinician perspectives on the usability of the hospital electronic medical record: A qualitative analysis

**DOI:** 10.1177/18333583231154624

**Published:** 2023-03-03

**Authors:** Sheree Lloyd, Karrie Long, Yasmine Probst, Josie Di Donato, Abraham Oshni Alvandi, Jeremy Roach, Christopher Bain

**Affiliations:** 1 Australian Institute of Health Service Management, 3925 University of Tasmania, Hobart, TAS, Australia; 2 90134 The Royal Melbourne Hospital, Parkville, VIC, Australia; 3 8691 University of Wollongong, Wollongong, NSW, Australia; 4 1969 Queensland University of Technology (QUT Online), Brisbane City, QLD, Australia; 5 StrongRoom AI, Docklands, VIC, Australia; 6 2541 Monash University, Melbourne, VIC, Australia

**Keywords:** computerised medical record systems, qualitative, electronic medical record, text analytics, usability, clinician, health information management

## Abstract

**Background:**

Electronic medical records (EMRs) have been widely implemented in Australian hospitals. Their usability and design to support clinicians to effectively deliver and document care is essential, as is their impact on clinical workflow, safety and quality, communication, and collaboration across health systems. Perceptions of, and data about, usability of EMRs implemented in Australian hospitals are key to successful adoption.

**Objective:**

To explore perspectives of medical and nursing clinicians on EMR usability utilising free-text data collected in a survey.

**Method:**

Qualitative analysis of one free-text optional question included in a web-based survey. Respondents included medical and nursing/midwifery professionals in Australian hospitals (85 doctors and 27 nurses), who commented on the usability of the main EMR used.

**Results:**

Themes identified related to the status of EMR implementation, system design, human factors, safety and risk, system response time, and stability, alerts, and supporting the collaboration between healthcare sectors. Positive factors included ability to view information from any location; ease of medication documentation; and capacity to access diagnostic test results. Usability concerns included lack of intuitiveness; complexity; difficulties communicating with primary and other care sectors; and time taken to perform clinical tasks.

**Conclusion:**

If the benefits of EMRs are to be realised, there are good reasons to address the usability challenges identified by clinicians. Easy solutions that could improve the usability experience of hospital-based clinicians include resolving sign-on issues, use of templates, and more intelligent alerts and warnings to avoid errors.

**Implications:**

These essential improvements to the usability of the EMR, which are the foundation of the digital health system, will enable hospital clinicians to deliver safer and more effective health care.

## Background

Poor usability of electronic medical and health records (EMRs and EHRs) has been documented as contributing to medical and nursing clinician fatigue, errors and burnout (Downing et al., 2018; Kaihlanen et al., 2020; Melnick et al., 2019). However, the use of EMR systems can also improve the quality, safety and efficiency of health services (Keasberry et al., 2017; Menachemi and Collum, 2011), and have been adopted to support clinical practice in the hospitals of advanced economies, such as Australia. As the implementation of EMRs progress, it is critical we understand the usability experiences of clinicians. In a post-pandemic world, much has changed; complexity and work pressures have intensified leading to a decline in wellbeing and loss of clinical staff from healthcare settings (Willis et al., 2021). The use of EMR systems that support clinicians to do their work without contributing to further burden is imperative. According to the International Standards Organisation (2018), usability comprises the aspects of effectiveness, efficiency, and satisfaction and represents the extent to which a product can be used to achieve specified goals in particular environments.

Globally, the terms EHR and EMR are often used interchangeably. In Australia, EHR is used to refer to the longitudinal health record summary (MyHealthRecord), while EMR is the organisation-based record used in hospitals, primary care and other health settings. A foundational EMR includes functionality to document patient histories, treatments, follow-up plans and to communicate with others providing care. A systematic review by Thomas-Craig et al. (2021) found that to mitigate against digital tools contributing to clinician burnout ‘careful examination of usability, introducing technologies to save or optimise time, and applying quality improvement to workflows’ (p. 985) is needed. Ratwani et al. (2018) identified that poor usability of digital tools, such as the EMR, shapes clinicians’ satisfaction with the systems. Nurses and medical clinicians are impacted by deficits in EMR usability. Researchers in Finland (Heponiemi et al., 2018; Kaipio et al., 2020; Vainiomäki et al., 2017) found patient safety could be endangered by technology-induced errors arising from EHR use for routine clinical work; and Kaihlanen et al. (2020) found that while poor usability of EMRs led to stress in both younger and older nurses, young nurses would benefit from systems that support them in completing routine tasks. Vehko et al. (2019) showed that the unreliability and poor user-friendliness of EMRs were prominent sources of psychological stress and time pressure among registered nurses. In the United States of America (USA), Melnick et al. (2020) found an association between EHR usability and workload among physicians, with favourable usability associated with reduced workload.

In Australia, while an increasing number of hospitals now use EMRs, these EMRs are at various stages of adoption, with each state health department and each private hospital adopting their own approach to implementation (McDonald, 2020). Although a range of products is available, market share has been dominated by a small number of vendors (McDonald, 2020). Levels of adoption and maturity of EMR systems differ and few Australian hospitals have achieved the highest stage of maturity validation as measured by the Healthcare Information and Management Systems Society (HIMSS) Electronic Medical Record Adoption Model (i.e. EMRAM Level 7) (HIMSS, 2020).

While research has demonstrated the impact of EMR usability on the wellbeing of clinicians (Downing et al., 2018; Melnick et al., 2020), little is known about usability perceptions of hospital-based clinicians across multiple Australian states. Given the global interest in EMR usability and the potential benefits of digitally enabled healthcare technologies, it is essential to understand perspectives of clinicians on EMR usability and the impact of EMR systems in hospitals.

The current study was part of a larger observational, cross-sectional survey conducted by Lloyd et al. (2021), which included the National Usability-Focused Health Information Systems Scale (NuHISS), developed and validated by Finnish researchers to collect national data on usability perceptions in Finland (Hyppönen et al., 2019; Kaipio et al., 2017, 2020), and deployed using a web-based tool (LimeSurvey). Additional to 13 questions capturing perceptions of EMR usability, the survey concluded with one optional, open-ended, free-text item to comment on the usability of the EMR used in their hospital. This open-ended question formed the basis for the current study. The aim was to explore the perspectives of hospital clinicians in relation to EMR usability by analysing the free-text data collected in the earlier survey.

## Method

### Sample and data collection

Medical, nursing and midwifery clinicians working in hospitals in Australia were recruited by invitation, using a purposive sampling approach (Gray, 2014). Invitations to participate were emailed to 34 professional bodies representing the health clinician disciplines who worked in the Australian healthcare system. A secondary snowball strategy used social media platforms (LinkedIn, Twitter, Facebook) to promote the survey. The member base of the Australasian Institute of Digital Health (AIDH) was also invited to participate. The survey was launched in June 2020 (following the first COVID-19 pandemic wave in Australia) and it remained open for 12 weeks. Participation was voluntary, participant information was provided, and consent obtained from respondents. Ethics approval was granted by Griffith University Human Research Ethics Committee (GU Ref No:2019/749). Findings from this survey have previously been reported (Lloyd et al., 2021).

### Data analysis

The free-text comments were deidentified and read independently by two members of the research team (SL and KL) before uploading to NVIVO for coding (Bazeley and Jackson, 2013). Responses to the open-ended item were separately coded by SL and KL to the four domains of usability of the NuHISS survey: ease of use; technical quality; benefits; and collaboration. When further phrases for coding were identified these were added in NVIVO. Where differences were identified between clinical coders, they were noted and discussed to achieve consensus. From the data, high-level themes and sub-themes related to usability were derived. Survey participants were invited to enter the EMR system they ‘mainly used’ in their clinical work, and could include the EMR brand and vendor, if known. This item was not compulsory.

## Results

### Demographic data

Of 143 medical respondents to the usability survey who worked in hospitals, 59% (*n* = 85) wrote comments in the free-text item. For nursing and midwifery, almost 50% (*n* = 27, 48%) of the 56 respondents provided additional comments before concluding the survey. The comments provided were brief and the complete data set was 5492 words.

Table S1 (online supplement) shows demographic characteristics for respondents who provided free-text comments: 65.9% (*n* = 56) of medical staff were <55 years of age, with the remainder (34.1%; *n* = 29) being 55 years or older; 51.8% (*n* = 14) of nurses and midwifery respondents were <55 years, with the remainder (*n* = 13) being 55+ years; 64.7% of medical staff and 62.9% of nurses and midwifery respondents were from Victoria; 24.7% of medical staff and 18.5% of nurses and midwifery respondents were from NSW; 61% (*n* = 52) of medical staff and 55% (*n* = 15) of nurses and midwifery respondents had >3 years’ experience with the EMR system they had used (or mainly used) at their hospital. One EMR brand was the most commonly used by the medical, nursing and midwifery clinicians (hereafter referred to as clinicians) who completed the free-text comment.

### Themes

Five themes and 11 sub-themes related to EMR usability were identified as shown in Table 1. *Status of EMR implementation* (Theme 1) influenced usability experiences with two sub-themes: the hybrid record (paper and electronic); and multiple logins and sign-outs. *Human factors and design of EMR* (Theme 2) impacted usability with four sub-themes: support for clinical workflows; searchability and information finding; lack of intuitiveness/learnability; and user interface design. *Safety and risk* (Theme 3), related to the benefits of EMRs, found three usability sub-themes: alerts and warnings; the ability of the EMR to create new errors; and cut/copy and paste functionality resulting in ‘note bloat’. *System response time and stability* (Theme 4) was reported as influencing usability with the sub-theme of delays, freezing and crashing. Usability issues related to *supporting collaborative care* (Theme 5) regarding communication and collaboration with external care providers (sub-theme extra-organisational communication).

**Table 1.** Themes and sub-themes related to domains of usability.

Themes	Sub-themes
Implementation	1.1 Hybrid records
1.Status of implementation of EMR	1.2 Multiple logins and sign-outs
Ease of use	
2.Human factors and design of EMR	2.1 Support for clinical workflows
	2.2 Searchability and information finding
	2.3 Lack of intuitiveness/learnability
	2.4 User interface design
Benefits	
3. Safety and risk	3.1 Alerts and warnings
	3.2 New error creation
	3.4 Note bloat
Technical quality	
4. System response time and stability	4.1 Delays, freezing and crashing
Collaboration	
5. Supporting collaborative care	5.1 Extra-organisational communication

#### Theme 1: Status of EMR implementation

Free-text comments from the clinicians reflected the status of EMR implementation in Australia at the time of the survey (early 2020). Respondents identified issues with hybrid (scanned and digital combined) records, multiple sign-ons for security, and ‘in progress’ implementations where the full suite of modules may not yet have been adopted in the user site.

##### Hybrid records

Clinicians’ impressions of the hybrid record and the use of multiple systems are illustrated in the following quotes: ‘Is slow and not intuitive. Is also not a fully paperless system - opd requests/consents and so on still need to be in a folder with patients’ (Nurse #420); ‘Main limitations are due to partial implementation and partial paper and partial electronic workflows’ (Medical #292); and ‘Key problem in my organisation is that there are multiple systems used in parallel and very hard to find information - often have to search across a combination of paper record/scanned medical record AND my department’s specific EHR’ (Medical #570).

##### Multiple logins and sign-outs

Reflecting the extent of progress of digitisation in Australia, some organisations have implemented a range of products from different vendors and may have not yet employed a single log in. Sign-out from systems to protect health information resulted in the requirement to regularly log in. These issues challenged users and led to a loss of productivity through wasted time and is exemplified in the quotes: ‘We still have different logins for different providers that means we waste time logging in etc.’ (Medical #483); ‘If you are not using the program, it shuts down so [you] have to repeatedly sign in throughout the day. While sitting at desk where I am using the program it will shut down. IT [Information Technology] department will not extend the time it is open. This makes the program very difficult and user unfriendly’ (Nurse #150); and ‘Loss of productivity. Takes 15 min to log in to all the necessary electronic systems and I can out-type the computer every time so need to slow down my typing to avoid errors’ (Medical #528).

#### Theme 2: Human factors and design of EMR

Comments from the clinicians related to human factors and design of the EMR systems used. EMR support for clinical workflow was a key theme. Clinicians also recognised that the EMR highlighted existing deficiencies in workflows and data capture.

##### Support for clinical workflows

‘The current systems require the clinician to change his [or] her workflow/practice/processes to accommodate the system, rather than the system accommodating the user’ (Medical #182);

The inability to easily use multiple inputs (e.g. handwriting, speech recognition etc.) and inability to easily search/scroll through records in a timely fashion has resulted in slowing down of clinic appointments, such that we now see half as many patients in the same amount of time that we did with paper records (Medical #182).

Comments suggested that the EMR slowed down the clinicians, with tasks taking longer than expected and detracting from patient-centred care. Nurses #324 and #225, respectively, noted that the EMR ‘slows down clinical care, detracts from interaction with the patients and it is slow and clunky and slows down staff work and takes away from patient engagement. (Nurse #225*)*; ‘increases time; decreases vigilance’ (Medical #279); and ‘everything takes longer and is slower from the clinician point of view because many of the tasks are purely clerical (typing my own letters, making appointment requests, deciding, and then making billing requests’ (in a public hospital) (Medical #117).

##### Searchability and information finding

Searchability and difficulty in locating information, a limitation of paper systems was also apparent in the EMR systems used. ‘[It] lacks a TAG system for facilitation of rapid location of notes by specific specialty, (especially) when searching previous admissions’ (Medical #388); ‘It is almost impossible to find the GP name and address. It should appear in the banner bar’ (Nurse #418); ‘Its search function leaves a lot to be desired’ (Medical #104); ‘Not searchable. I think this is really important’ (Nurse #225); ‘Information can easily get lost. Different staff using different screens cannot see other screens where important information might be entered. My work is 30% slower since (Brand B)’ (Medical #293); and ‘Finding radiology images is time consuming and slow, there are too many buttons that need to be pressed to pull up an X-ray. Ditto for respiratory function tests and sleep study reports’ (Medical #578).

##### Lack of intuitiveness and learnability

This sub-theme highlighted that intuitiveness of the EMR systems for clinicians was low. The lack of intuitiveness of the EMR system made it difficult to learn and necessitated the need for training. Training can be challenging with a highly mobile workforce, locum staff, pool nurses and casuals all required to use and access EMRs. ‘Non-intuitive. So many steps to complete one task’ (Nurse #340); ‘Not intuitive. Training required and not practical for a mobile/dispersed/sessional workforce’ (Medical #549); ‘Complex. Not intuitive. Difficult to follow. No training and little support’ (Medical #476).

##### User interface design

Clinicians reported features of EMR usability concerning the design of the user interface. Screen fonts and layout, consistency, and visibility of actions impact upon the user experience and sentiments are reflected in exemplar quotations: ‘User interface is dated and inconsistent from screen to screen. Important option menus are hard to find because they are in small font. It is not intuitive’ (Nurse #523);

there are lots of unused icons which just confuse the UX [User Experience]. Also, the font sizes are fixed and as someone with deteriorating vision, I struggle to read some of the information that is presented. Also, some of the font choices are questionable. (Medical #578).

A desire for flexibility of the system and tailoring of EMRs to individual user’s requirements and support for efficiency of use (reduced clicks and shortcuts) was described: ‘Inability to set own default settings. I provide a consultation service so am interested in observations done over the past week not 24 h. However, I can’t set this up on the EMR for when I log into it’ (Medical #336); ‘Less clicks to achieve what you need. More shortcuts for reducing time on repetitive tasks’ (Medical #566);

Greater appreciation of human factors should be incorporated into their design and implementation, and a participatory design methodology should be employed to ensure that the end-user is actually involved in the process. “Click conservation” should also be considered. It shouldn’t take 10 clicks to do something that can be done in two or three. (Medical #496).

#### Theme 3: Safety and risk

Clinicians reported on benefits of EMRs that can be attained by reducing errors, improving legibility, ease of access to results, error warnings and medication safety. Exemplar quotations demonstrated: ‘Easier to do medication documentation and prescribing but must be regularly checked and updated’ (Medical #117); ‘Good things about EMR are accessing results and imaging are now easier’ (Medical #117); ‘Improved processes with EMR - legibility and remote access to prescribing’ (Medical #388).

##### Alerts and warnings

The clinicians commented on warnings, handling of allergies and medication vulnerabilities due to EMR system availability. When describing warnings, medical participants related that: ‘Allergies on (anaesthetics module) as well as (Brand C module EMR) show drugs but not the severity of allergy on the main page: if a patient has life threatening anaphylaxis, it appears no different on screen compared to someone who gets constipation or minor side effects. This is a major fault’ (Medical #620); when speaking about the e-medication system: ‘The automatic warnings are silly, whereas there are no warnings for duplicate orders, including opioids and paracetamol’ (Medical #620).

##### New error creation

The ability of the EMR to contribute to or create new errors was of concern to medical and nursing respondents.

Minor differences in build and policies between domains means staff moving between LHDs [Local Health Districts] do not necessarily understand the subtle differences and make errors based on assumptions of how it worked in their previous place of employment (Nurse #212) and creates new errors that are difficult to detect. (Medical #561)

##### Note bloat

‘Note bloat’ due to cut/copy and paste was identified by some clinicians, represented in the following quotes: ‘There is a tendency for junior (and senior) staff to cut and paste which means often wrong information is perpetuated’ (Medical #117); ‘Copy and paste replacing documentation of clinical reasoning is rife. It is hard to find relevant clinical notes among the many. Cross referencing is poor’ (Medical #327);

The “cut n paste” practice of JMO can lead to promulgation of inaccuracies. Rapid scanning of notes for entries by specific groups/persons is hampered by the homogeneous font and color. Senior docs come to rely on JMO to document the ward round. They miss much of the subtle detail, which I could have written myself, if I was writing on paper at the bedside. Tasks of writing notes & prescribing cannot be performed in parallel, so can be much less time efficient when performed sequentially. (Medical #388).

#### Theme 4: System response times and stability

This theme and sub-theme related to clinicians experiencing slow responses to data entry, data loss and challenges when EMR systems crashed or were not available.

##### Delays, freezing and crashing

Response times and stability of the EMR system used revealed a sub-theme of delays, freezing and crashing, as exemplified in the following quotes. ‘Crashes occasionally. Freezes often’ (Medical #522); ‘Biggest problem is that it's slow, and not intuitive’ (Medical #651); ‘Frequently multiple ways of attempting a task only for application to either crash or experience a script error’ (Medical #525) and ‘the system sometimes loses data or does not receive data’ (Medical #620). ‘Brand A can be slow with lag time between clicking on a task and the task being processed’ (Medical #508); and ‘The EMR system has crashed for several days on 2 separate occasions & I have felt vulnerable to possible medication errors occurring’ (Nurse #423).

#### Theme 5: Supporting collaborative care

Communication within the hospital, across disciplines and teams to support the delivery of high-quality and safe care is critical. Positive commentary such as access to records when not on the hospital site that can support anywhere, anytime care and advice to the hospital care team was valued as reflected in example quotations. ‘Checking up on what has been recorded from a remote location’ (Medical #388); and a medical clinician related that ‘the ability to access patient notes remotely is extremely helpful’ (Medical #270).

##### Extra-organisational communication

Communication between clinicians so that information collected in primary and other sectors is available to inform care when needed was a benefit noted by clinicians as well as access to patient’s My Health Record, Australia’s digital health information summary. Comments suggested that there are still challenges with the EMR as a tool to support patient care across and between different sectors and expressed in exemplar quotations: ‘Lots of great things about EMR and it is an improvement on writing but should be standardised across the state and indeed Australia so someone’s medical record can be reviewed from anywhere’ (Medical #513); and ‘now that it's linking some patients' hospital records with their My Health Record, we can find some documents easier that come from external providers’ (Medical #483); ‘Poor interoperability with aged care, primary care, and in-hospital systems’ (Medical #549); and ‘poor linkage with primary care, no flexibility for our patient type processes such as dialysis, poor flexibility, and responsiveness to change’ (Medical #106); ‘difficult to audit and takes such a lot of effort to find external scanned documents, for example, GP and specialist letters, ECGs done elsewhere’ (Medical #290). Finally, participants remained hopeful for the potential of EMR systems to support the continuum of care: ‘I think there is potential for a way more user friendly and better integrated system for patients and across different health providers from the community to the hospital and back’ (Medical #451).

## Discussion

Our study reflects the perspectives of participant clinicians on EMR usability and the current status of implementation in Australian hospitals. Levels of adoption and maturity of EMR systems differ and few Australian hospitals have achieved EMRAM Level 7, with all ancillary systems, a complete EMR with clinical decision support and data analytics implemented (HIMSS, 2020). Some usability issues identified in the study could be due to the clinicians’ use of hybrid record systems, as other studies have reported (Magrabi et al., 2016; Schiff et al., 2015). This aside, pertinent insights on EMR usability were gathered from experienced clinicians in our study, (reflected by the age and years of experience of those who provided comments).

Research data describing clinician perceptions of EMR usability from Australian hospitals across multiple sites is scarce, and this is the major contribution of this study. Clinicians in our study reported that the EMR disrupted clinical workflows and increased the time taken to complete routine tasks, such as documentation. This finding concurs with the systematic review by Keasberry et al. (2017), and other studies that describe time burden of EMRs (Downing et al., 2018; Dymek et al., 2021; Rizvi et al., 2017). Further, a review of factors related to successful EMR implementation by Fennelly et al. (2020) found the ‘inability of the EHR system to meet the workflows of end users and organisations was commonly cited as negatively impacting on success’ (p. 11). These challenges to clinical workflow could be addressed through the adoption of evidence-based techniques that leverage speech recognition technologies, natural language processing, artificial intelligence and redesign of EHR workflow (Dymek et al., 2021); and enhancements to software products (e.g. EMR inbox management optimisation) and could improve clinician user experience (American Medical Association, 2022; Dymek et al., 2021; Murphy et al., 2019). The introduction of EMR templates has also been demonstrated to reduce note bloat (Kahn et al., 2018).

Technology and software solutions that support single and less frequent need to sign in to hospital applications could be a simple solution in a ‘time poor’ clinical environment. Single sign-on software and/or proximity card logon or biometric identification can support the process or reduce the frequency of sign-on (Flores-Zuniga et al., 2010; Fontaine et al., 2016; Gellert et al., 2017; Vainiomäki et al., 2017). For example, to reduce the number of ‘clicks’ required in the EMR, a study by Guo et al. (2017) demonstrated that physician-driven changes to assist documentation, ordering and chart review reduced the click burden and resulted in improved workflows. This was achieved by physicians collaborating with the vendor and the hospital IT Department. Tailoring EMR systems to assist with performing clinician's routine clinical tasks and to complement their workflows would be advantageous (Horsky and Ramelson, 2016; Neri et al., 2015; Ommaya et al., 2018; Vainiomäki et al., 2020).

EMR system complexity and lack of intuitiveness, coupled with a highly mobile clinician population may lead to an underutilisation of EMR system functionalities, potentially undermining the accuracy and integrity of data collected (i.e. cut and paste; note bloat), and the creation of new errors (Ommaya et al., 2018; Scott et al., 2018; Sittig et al., 2016). EMR training that demonstrates clinician user shortcuts, optimal use and approaches that shift from the traditional classroom teaching to techniques such as interactive and workflow-based content and hands-on rehearsals in simulated work environments may prove more effective (Scott et al., 2018; Ting et al., 2021).

EMR system stability and response times influence usability (Nielsen, 1993; Vainiomäki et al., 2017) and remediation requires an investment in IT and communication infrastructure, server upgrades, software enhancements and other resources to address the causes of technical instability and delay. According to the clinicians in our study, difficulties with sharing information between and across health sectors remain; and in spite of concerted efforts and a focus on strengthening information sharing in Australia (through MyHealthRecord and other initiatives) this benefit has not yet been fully realised. In Australia, there is a complicated system of funding, accountability for, and delivery of, healthcare systems (Duckett and Willcox, 2015). Solving accompanying challenges is complex and implementation of EMRs in each sector has been performed in isolation. Challenges with interoperability are related to a range of issues, including standards (Rowlands, 2020), and the Australian Digital Health Agency continues to progress work to move this forward toward an effective and interoperable future (Australian Digital Health Agency, 2018, 2021).

Concerns about safety and risk, allergy and medication alerts, and warnings identified by clinicians have pointed to usability, software design, and/or issues with EMR functionality (Kauppinen et al., 2017; Ratwani et al., 2018). These safety, workflow, communication and interoperability findings concur with a state-wide study of nurses in Texas, which examined qualitative comments relating to EMR experiences in clinical settings (McBride et al., 2017). The creation of new errors introduced with the EMR was identified by Hecht (2019); and alert types, meaningfulness and frequency has been reported to result in desensitisation through alert fatigue and inbox burden in studies by Ancker et al. (2017) and Yan et al. (2021). Schubel et al. (2020) urged users (i.e. clinicians), developers and designers to work together to improve alert management. Addressing these issues in the USA, Ratwani et al. (2017) argued that EMR vendors achieve safety-enhanced design (SED) certification requirements. Addressing the safety and alert issues described by clinicians in our study should be a priority.

EMRs can be presented to hospital clinicians as the solution for many ills, but there is an expectation and experience gap between promised benefits and their interactions with systems. Clinicians have experience and knowledge of the way technologies are being used outside of the hospital setting, at home, and by other industries (banking, leisure, governments) and embrace the potential they offer. Crowley et al. (2019) suggested that when acquiring new systems, workforce communication approaches should balance potential expected benefits with realistic expectations. The benefits and timeframes for attaining them should be specific to the organisation and communicated clearly to end users (Fennelly et al., 2020).

When acquiring EMR systems, applying evaluation processes such as heuristic evaluation, accompanied by additional measures, may avoid some of the usability issues currently experienced (Khajouei et al., 2018; Nielsen, 2020; Savoy et al., 2017). In a rapid umbrella review that investigated implementation of a national electronic health record, the reengineering of workflows for efficiency, testing for usability and involvement of users throughout all stages were observed as key success factors (Fennelly et al., 2020). Further, Scott et al. (2018) provided a checklist for successful EMR implementation, including guidance on the elements of the ideal user interface. When acquiring EMR systems, purchasers in Australia can apply frameworks to measure the safety and usability of systems (Ratwani et al., 2017). Purchasers require a comprehensive understanding of the practices and progress that vendors are making to address usability in their EMR systems (Hettinger et al., 2021; McBride et al., 2017; Sittig et al., 2020). Vendors need to demonstrate that they are applying human-oriented design approaches to systems and interfaces that cater for various levels of users (expert to beginner), support clinical workflows, and that are usable, desirable and technically feasible (Cooper et al., 2014).

The clinical workforce is a critical resource in Australian hospitals. It is recognised that EMRs are an important tool in the hospital for documentation and an appreciation that health information systems can contribute to nurse and physician stress is imperative. Our challenge is to address the usability factors that contribute to this, such as inefficiently designed user interfaces, inadequate health information exchange with external organisations and excessive data entry requirements (Downing et al., 2018; Kroth et al., 2018; Melnick et al., 2020; Vainiomäki et al., 2017, 2020).

### Limitations

Our qualitative data were responses from participating hospital clinicians to one free-text, open-ended question included as part of a larger survey. While nursing and midwifery clinicians encompass the largest workforce in Australian hospital, the majority of participant comments in this study were from medical clinicians; and most comments were from users of one EMR brand. Further exploration utilising interviews and focus groups could elicit more detailed and in-depth data, and could lead to new concepts and ideas to improve the usability of EMRs in hospitals.

This study was conducted during 2020, with data collection affected by the COVID pandemic that disrupted the health system and added burden to the hospital system in most Australian states. This may have caused a relative overestimation of usability issues reported by the clinicians using EMRs. However, evidence from other countries suggests that the issues raised by the clinicians in our study are similar and pre-date the pandemic (Downing et al., 2018; Kaipio et al., 2017; Melnick et al., 2020).

## Conclusion

Our study found that clinicians working in hospitals experienced a variety of usability concerns, including hybrid record systems, multiple logins, poor integration of the EMR with clinical workflows, limitations in communicating with external care providers, ‘note bloat’ due to copy and paste, potential for new error creation, alerts and warnings, system response time and stability, and lack of intuitiveness/learnability. If the benefits of EMRs are to be realised there are good reasons to address the usability challenges identified by clinicians. There are solutions to improve the usability experience of hospital-based clinicians, such as resolving sign-on issues, use of templates, and more intelligent alerts and warnings to avoid errors. Improvements to the usability of the EMR system are essential as they are the foundation of the digital health system. A strong foundation that collects quality data, averts errors, prevents adverse events, and supports clinicians to provide care and collaborate with colleagues will enable us to move to the next level of digital health transformation and to attain the benefits of patient-centred care. The challenge is for all stakeholders (clinicians, vendors and health IT developers) to implement from the evidence base, proven strategies, and solutions to optimise usability to strengthen EMR systems and hospital-based clinicians user experience. Purchasers should understand vendor approaches to human factors and user-centred design; while vendors and purchasers should focus attention on EMR usability issues such as error creation and near misses for medications.

## Supplemental Material

Supplemental Material - Medical and nursing clinician perspectives on the usability of the hospital electronic medical record: A qualitative analysis Supplemental Material for Medical and nursing clinician perspectives on the usability of the hospital electronic medical record: A qualitative analysis by Sheree Lloyd, Karrie Long, Yasmine Probst, Josie Di Donato, Abraham O Alvandi, Jeremy Roach, and Christopher Bain in Health Information Management Journal
