# Supplementary material for: Medical and nursing clinician perspectives on the usability of the hospital electronic medical record: A qualitative analysis
Source: Health Inf Manag. 2023 Mar 3;53(3):189–97. doi: 10.1177/18333583231154624 (PMC11401339; doi:10.1177/18333583231154624)
Supplement: Supplemental Material - Medical and nursing clinician perspectives on the usability of the hospital electronic medical record: A qualitative analysis [file sj-pdf-1-him-10.1177_18333583231154624.pdf]

Table S1: Participant clinician demographic data

	N (% of total respondents)	
	**Medical clinicians**	**Nurse/midwifery clinicians**
**Age**		
25 - 34 years	5 (5.9)	2 (7.4)
35 - 44 years	26 (30.6)	12 (44.4)
45 - 54 years	25 (29.4)	6 (22.2)
55 - 65 years	24 (28.2)	7 (25.9)
65 years and over	5 (5.9)	0
**Total**	**85 (100)**	**27 (100)**
**Gender**		
Female	36 (42.3)	20 (74)
Male	47 (55.2)	6 (22.2)
Prefer not to say	2 (2.4)	1 (3.7)
**Total**	**85 (100)**	**27 (100)**
**State**		
NSW	21 (24.7)	5 (18.5)
QLD	2 (2.3)	3 (11.1)
SA	3 (3.5)	2 (7.4)
VIC	55 (64.7)	17 (62.9)
WA	3 (3.5)	0
Not stated	1 (1.2)	0
**Total**	**85 (100)**	**27 (100)**
**Years of experience with EMR mainly used**		
< 12 months	10 (11.8)	7 (25.9)
1-3 years	22 (25.8)	5 (18.5)
3-6 years	20 (23.5)	7 (25.9)
More than 6 years	32 (37.6)	8 (29.6)
Not stated	1 (1.2)	0
**Total**	**85 (100)**	**27 (100)**
